# Advancing the interfacing performances of chronically implantable neural probes in the era of CMOS neuroelectronics

**DOI:** 10.3389/fnins.2023.1275908

**Published:** 2023-10-31

**Authors:** Alberto Perna, Gian Nicola Angotzi, Luca Berdondini, João Filipe Ribeiro

**Affiliations:** ^1^Microtechnology for Neuroelectronics Lab, Fondazione Istituto Italiano di Tecnologia, Neuroscience and Brain Technologies, Genova, Italy; ^2^The Open University Affiliated Research Centre at Istituto Italiano di Tecnologia (ARC@IIT), Istituto Italiano di Tecnologia, Genova, Italy

**Keywords:** brain-computer interfaces, chronic implants, CMOS neural probes, intracortical electrodes, Foreign Body Reaction, bending stiffness, implantation procedure, surface physicochemical properties

## Abstract

Tissue penetrating microelectrode neural probes can record electrophysiological brain signals at resolutions down to single neurons, making them invaluable tools for neuroscience research and Brain-Computer-Interfaces (BCIs). The known gradual decrease of their electrical interfacing performances in chronic settings, however, remains a major challenge. A key factor leading to such decay is Foreign Body Reaction (FBR), which is the cascade of biological responses that occurs in the brain in the presence of a tissue damaging artificial device. Interestingly, the recent adoption of Complementary Metal Oxide Semiconductor (CMOS) technology to realize implantable neural probes capable of monitoring hundreds to thousands of neurons simultaneously, may open new opportunities to face the FBR challenge. Indeed, this shift from passive Micro Electro-Mechanical Systems (MEMS) to active CMOS neural probe technologies creates important, yet unexplored, opportunities to tune probe features such as the mechanical properties of the probe, its layout, size, and surface physicochemical properties, to minimize tissue damage and consequently FBR. Here, we will first review relevant literature on FBR to provide a better understanding of the processes and sources underlying this tissue response. Methods to assess FBR will be described, including conventional approaches based on the imaging of biomarkers, and more recent transcriptomics technologies. Then, we will consider emerging opportunities offered by the features of CMOS probes. Finally, we will describe a prototypical neural probe that may meet the needs for advancing clinical BCIs, and we propose axial insertion force as a potential metric to assess the influence of probe features on acute tissue damage and to control the implantation procedure to minimize iatrogenic injury and subsequent FBR.

## 1. Introduction

Implantable microelectrode array neural probes can record high quality neural signals with sub-millisecond temporal resolution from multiple neuronal cells. Consequently, these neuro devices have become invaluable tools to advance the study of complex brain circuits and the processes underlying brain diseases and neurological disorders, and for the development of diagnostic clinical instrumentation, brain-computer-interfaces (BCIs) and therapeutic electroceuticals (Maharbiz et al., 2017). Implantable probes provide a much greater spatial and temporal resolution to access fine grained neural activity when compared to non-invasive electroencephalography (EEG) devices (Abiri et al., 2019) and to less invasive electrocorticography (ECoG) devices (Schalk and Leuthardt, 2011), and thus provide a rich information content exploitable in BCIs. BCIs based on implantable electrode probes have already been successfully used in rats (Chapin et al., 1999), non-human primates (Wessberg et al., 2000; Carmena et al., 2003; Lebedev et al., 2005; Velliste et al., 2008; Willsey et al., 2022) and also in tetraplegic human patients (Hochberg et al., 2012; Handelman et al., 2022) to demonstrate the control over robotic arms with single and multiple degrees of freedom. Recently, the BrainGate team has presented BCIs that allow to restore communication in patients who have lost their ability to speak, either by decoding neural signals associated with handwriting (Willett et al., 2021) or by interpreting their intention to utter phonemes and words (Willett et al., 2023). This second approach allowed to decode attempted speech at the impressive rate of 62 words per minute, which is 3.4 times faster than previously reported date, and starts to approach the speed of a natural conversation (160 words per minute).

In this context, it is widely recognized that neural probes enabling large-scale recordings of neuronal signals in the brain are required to advance BCIs to restore motor control and communication capabilities (Nicolelis, 2001) in severely disabled patients, particularly those suffering from debilitating conditions such as amyotrophic lateral sclerosis, spinal cord injury, stroke and cerebral palsy (Lebedev and Nicolelis, 2006). Intracortical neural recording probes with high channel counts may therefore significantly contribute to the advancement of BCIs, such as the one recently introduced by Lorach et al. (2023), combining ECoG recordings and epidural electrical stimulation enabling a tetraplegic individual to regain the ability to walk.

**Figure 1:**
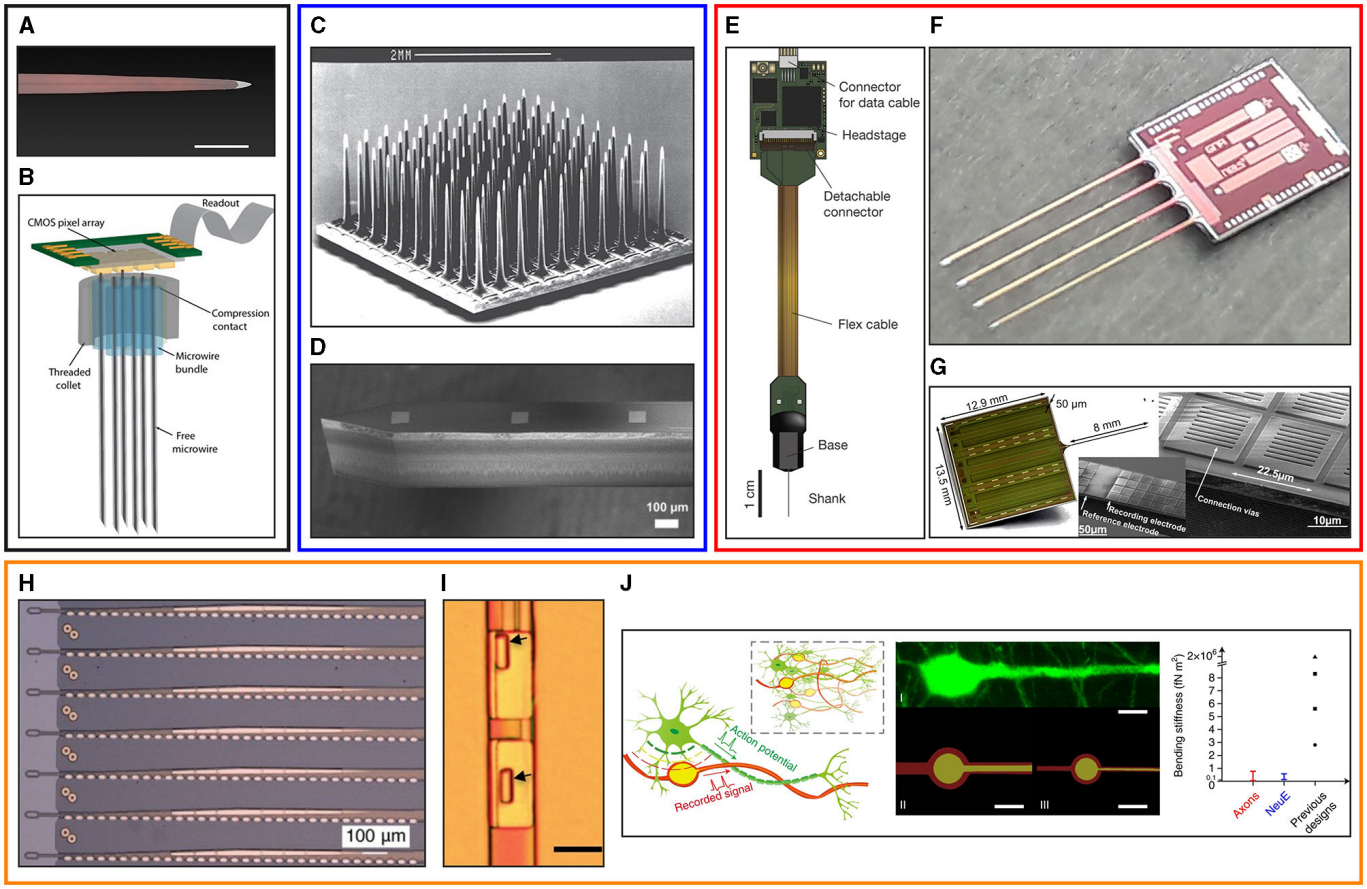
Overview of different types of intracortical microelectrode probes. Top left (black panel): microwire neural probes. **(A)** An individual microwire, the first technology used to routinely record neural signals from the brain (adapted from Winslow and Tresco, 2010, scale bar 100 μm) and **(B)** a bundle of microwires mated to a large-scale microelectrode array for signal acquisition (adapted from Obaid et al., 2020a). Top middle (blue panel): passive silicon probes. **(C)** The Utah array, a typical example of an out of plane silicon probe (adapted from Normann and Fernandez, 2016) and **(D)** a Michigan style silicon probe realized with the so called in plane processing (adapted from Fekete, 2015). Top right (red panel): active dense CMOS neural probes. **(E)** The Neuropixel probe (adapted from Jun et al., 2017), **(F)** the SiNAPS probe (adapted from Ribeiro et al., 2022) and **(G)** the NeuroSeeker probe (adapted from Raducanu et al., 2017). Bottom: (orange panel): flexible probes. **(H)** Electrode array fabricated on SU-8 substrate (adapted from Musk, 2019) **(I)** Nanoelectronic thread probes (scale bar 10 μm) realized with SU-8 substrate (adapted from Luan et al., 2017) and **(J)** bio-inspired neuron-like electronic flexible probes (adapted from Yang et al., 2019).

However, despite the widespread potential application of these devices, the use of tissue penetrating neural probes in a chronic implant setting faces multiple limiting factors, among which Foreign Body Reaction (FBR) is widely recognized as being of primary importance (Szarowski et al., 2003; Seymour and Kipke, 2007; McConnell et al., 2009). Numerous papers have underlined the impact of FBR on the chronic electrical performances of tissue penetrating neural probes, as reviewed by Tresco and Winslow (2011) and by Ferguson et al. (2019). These works point to the increase in the electrode impedance caused by glial encapsulation and to neuronal cell death as key (although not exclusive) factors for the decline in probe recording performances observed over time. These observations have motivated the neuroengineering community to study and develop different types of neural probes. As reported in Figure 1, implantable neural probes are currently distinguished into different categories: microwire based probes (Figures 1A, B), stiff passive probes (Figures 1C, D), stiff active probes (Figures 1E–G) and flexible neural probes (Figures 1H–J).

Among these categories, an initial distinction can be made between flexible and stiff devices. This distinction emerges from several studies that investigated the effects of the mismatch between the mechanical properties of the brain and of the substrate of neural probes, revealing this to be a key factor of enhanced stress at the biotic-abiotic interface, particularly for chronically implanted neural probes (Subbaroyan et al., 2005; Nguyen et al., 2014; Polanco et al., 2014, 2016). Further, the periodic micromotion of the brain due to physiological and behavioral sources can induce continuous strain and repeated injury on the soft brain tissue in contact with comparatively stiffer devices, ultimately leading to increased local inflammation and more severe FBR outcome (Gilletti and Muthuswamy, 2006). Lecomte et al. (2018) reviewed and analyzed mechanical interactions taking place between neural probes and brain tissue. In this work, the importance of mechanical probe features such as substrate bulk properties and overall bending stiffness is highlighted, as they are shown to play an important role in eliciting brain tissue responses.

Given the recent introduction of CMOS neural probes consisting of monolithic and micro-structured CMOS neuroelectronic chips, neural probes can currently also be separated into passive and active devices. Active neural probes were primarily developed to overcome limitations in the number and density of integrated microelectrode sensors typically provided by conventional, i.e., passive, neural probes realized using stiff silicon, or flexible polymeric substrates. These conventional passive probes integrate microelectrodes which are individually (passively) wired to contact pads used for interconnection with an acquisition instrument or for their hybrid integration with front-end CMOS chips (Musk, 2019; Zhao et al., 2023). Such electrode-to-pads routing of electrical interconnects on-chip is the main limiting factor for fabricating large and dense microelectrode arrays (Berdondini et al., 2009). However, active neural probes based on CMOS circuits such as Neuropixels (Jun et al., 2017), NeuroSeeker (Raducanu et al., 2017), and SiNAPS probes (Angotzi et al., 2019; Ribeiro et al., 2021) can overcome these limitations by embedding on-probe circuits for front-end signal conditioning, time-division multiplexing and addressing logic (Angotzi et al., 2017). By enabling access to neuronal spiking activity concomitantly recorded from dense arrays of microelectrodes located in multiple brain areas, this approach offers new opportunities to study mechanisms underlying the execution of brain functions and to investigate the root causes of neurological diseases, such as Parkinson and Alzheimer (Benabid et al., 2009; Jeon et al., 2014). It has to be noted that the technology used to produce active neural probes will also open up the possibility of creating smaller area, less invasive, implantable shanks with high channel count compared with the technology of passive (stiff or flexible) probes.

Based on the above premises, this review will focus on emerging strategies to face the challenge of achieving high-channel count, chronically stable neural probes. We will first discuss the physiological constraints determined by the biological mechanisms underlying acute and chronic tissue reactions to implanted neural probes, and report different approaches that can be used to assess FBR. Secondly, we will review the different factors relevant for the design and use of chronically implanted neural probes. Finally, following the description of the key features of active neural probes with respect to passive probes, we describe a prototypical device that aims to exploit these features to achieve high-quality, long-term and large-scale neuronal recordings for next-generation BCIs.

## 2. Physiological constraints

The design of chronically stable neural interfaces faces several physiological constraints that are described in detail below. These constraints are determined by a number of factors, namely, mismatch of mechanical properties between the neural probe and the brain, neurovascular and cortical damage induced by the implantation, possible infections due to the invasive nature of the implantation procedures, constant relative motion at the biotic-abiotic interface arising from physiological and behavioral sources, as well as physicochemical interactions taking place at the biotic-abiotic interface.

### 2.1. Brain mechanical properties

In order to understand the mechanisms underlying brain injury and the nature of acute and chronic interactions between the brain and a foreign body it is necessary to carry out an in-depth characterization of the mechanical properties of the brain tissue (Prevost et al., 2011). Brain tissue is an inhomogeneous, nonlinear and anisotropic viscoelastic material, which makes its mechanical characterization a challenging endeavor. The choice of an appropriate material model to represent it is not trivial, since its properties depend on the strain rate and on the type of applied load (tension, compression, shear, etc.). Furthermore, the brain is strongly inhomogeneous from a mechanical standpoint. White matter consists of highly oriented fiber arrangements, while gray matter is composed of cell bodies and the supporting vascular network. These architectural differences are reflected in the unalike mechanical properties observed in different brain regions. For example, the corpus callosum, a highly aligned, uniaxially oriented region of the brain is significantly anisotropic, while gray matter structures display a more isotropic behavior (Prange and Margulies, 2002). Moreover, several studies have reported white matter to be significantly stiffer than gray matter (Budday et al., 2015, 2020; Weickenmeier et al., 2016) and have determined a positive correlation between myelin content and brain stiffness (Weickenmeier et al., 2016). Most studies report a storage and loss shear modulus of brain tissue in the range from 1 to 10 kPa; however, results differ by an order of magnitude, even within the same research group, depending on experimental conditions. This variability is not only caused by the inherent heterogeneity in tissue properties due to age, sex, species, and other biological factors, but also by protocol choices and apparatus used to perform the mechanical measures (Cheng et al., 2008).

Tissue penetrating neural probes are generally implanted through the cortex and may reach different depths based on the length of the probe and on the targeted brain region. In the selection of the implantation site, care is typically taken to avoid superficial vasculature, particularly large blood vessels, to avoid excessive bleeding and BBB rupture that might lead to more severe FBR outcomes. Depending on the location and the depth of the implant, the device might cross several brain areas (e.g., corpus callosum, hippocampus, thalamus). These different structures have different morphological and mechanical features, but to the best of our knowledge there are no in-depth investigations on the impact of such heterogeneity on FBR and chronic probe performance.

An aspect which emerges from the literature as a driver for mechanical interactions between tissue penetrating neural probes and brain tissue is the strategy used to tether the device to the skull. Implants rigidly tethered to the skull were reported to increase FBR compared to free floating implants (Kim et al., 2004; Biran et al., 2007; Thelin et al., 2011; Chauviere et al., 2019), potentially because this fixation modality increases the magnitude of the relative micromotion among the probe and the brain tissue.

### 2.2. Brain micromotion

Relative motion between an implanted probe and the brain, arising from physiological (cardiac and respiration rhythm) or behavioral sources (spontaneous head/trunk displacements), causes shear stress at the biotic-abiotic interface, which is enhanced by the mismatch in mechanical properties (Gilletti and Muthuswamy, 2006; Lind et al., 2013).

**Figure 2:**
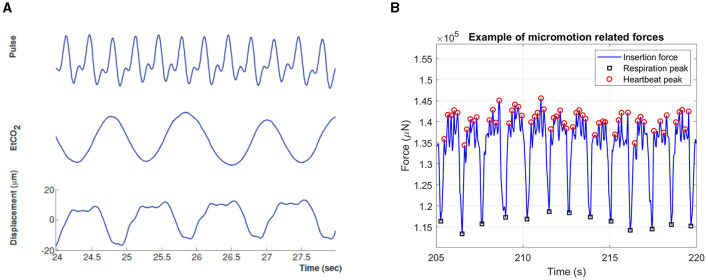
Physiological signals at the origin of micromotion. **(A)** Graph representing micromotion of the brain due to heartbeat and respiration. Cortical displacement correlates with electrocardiogram (cardiac pulsation) and end tidal CO2 (respiration) measurements. Adapted from Gilletti and Muthuswamy (2006). **(B)** Brain micromotion forces measured during the implantation of stiff tissue penetrating silicon probes. The peaks marked with black circles are due to the animal respiration, while the smaller ones highlighted with red circles are due to the heartbeat. Adapted from Perna et al. (2023).

Gilletti and Muthuswamy quantified the amplitude of surface micromotion in the rat brain and observed that respiration can induce 10–30 μm periodic displacements, while the amplitude of heartbeat-induced micromotion is in the range of 2–4 μm (Figure 2A) (Gilletti and Muthuswamy, 2006). During the implantation of stiff silicon probes, these displacements translate into measurable micromotion forces, as shown in Figure 2B.

The characterization of brain micromotion, allowed to develop a 3D *in-vitro* glial scar model in which primary brain cell cultures were subjected to axial micromotion with realistic amplitude and frequency (Spencer et al., 2017). In this model, the area and perimeter of astrocytes were found to increase significantly in response to micromotion, thus demonstrating the impact of chronic mechanical stress on glial reactivity. Other *in-vitro* models have also been developed to simulate the impact of cyclic mechanical loading on co-cultures of astrocytes, microglia and neurons. Here, chronic strain was observed to produce the upregulation of Interleukin receptor antagonist IL-36Ra, of matrix metalloproteinases 2 and 9 and of glial fibrillary acidic protein (GFAP), as well as neuronal cell death (Cullen et al., 2007; Karumbaiah et al., 2012). However, the loading conditions used in these models are less realistic compared to the first one, because the deformation is applied to the substrate on which the cells are grown rather than provided as an external shear stress.

The impact of micromotion at a cellular level has also been studied *in-vivo*; Sridharan et al. (2021) observed that micromotion at the probe-tissue interface induces changes in the membrane potential of nearby neurons, possibly through the activation of mechano-sensitive ion channels.

### 2.3. Foreign Body Reaction

The biological reaction that onsets in the presence of an implanted device, referred to as FBR, is a complex physiological process that impacts the chronic performances of neural microelectrode probes.

**Figure 3:**
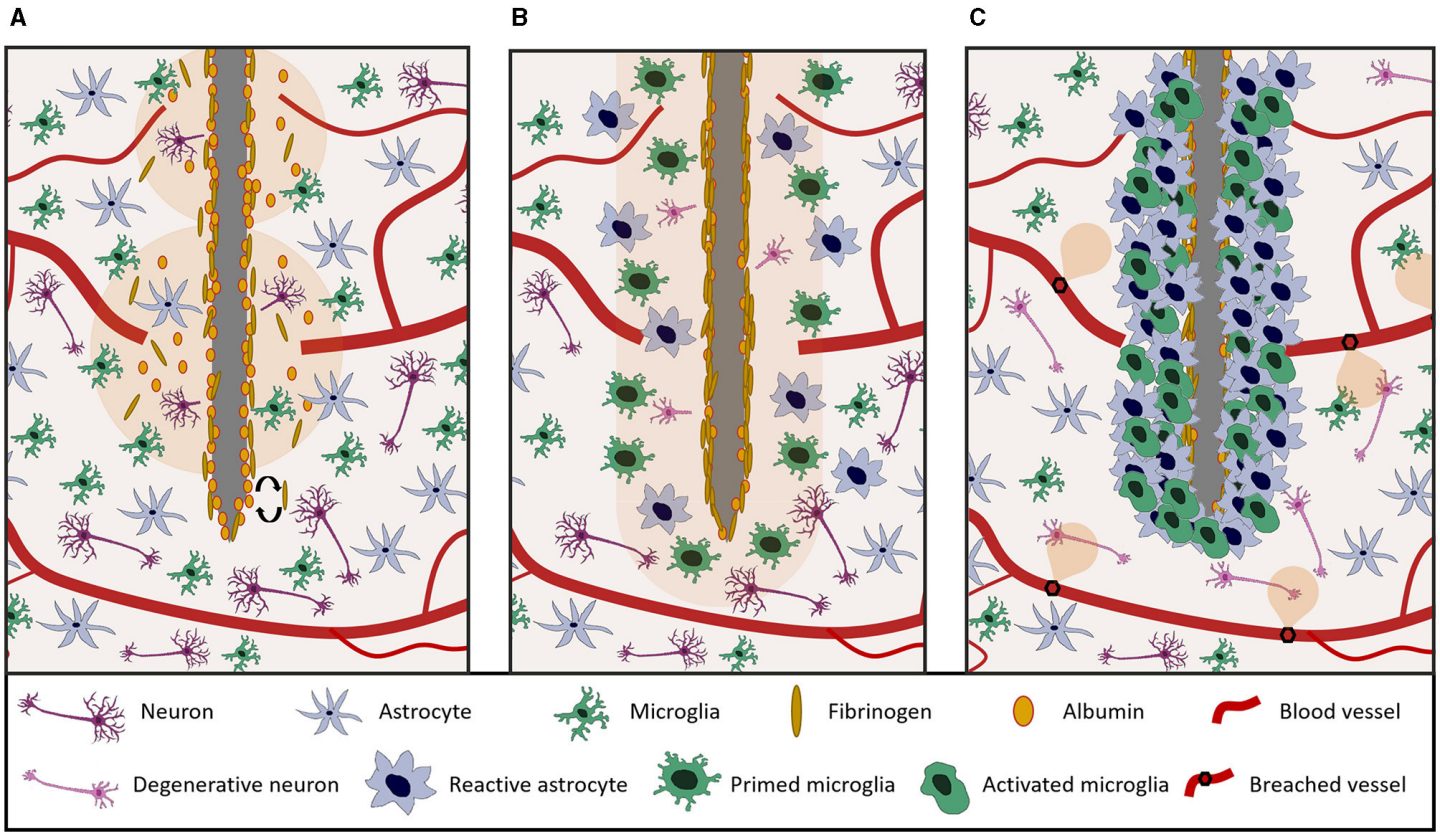
Overview of the main different stages of FBR to tissue penetrating neural probes. **(A)** Acute neurovascular and cortical damage at implantation. Probe insertion ruptures blood vessels causing plasma extravasation and severs neuronal bodies. **(B)** Short term (hours-days) response to probe implantation. Microglia and astroglia are activated and they begin to proliferate and migrate toward the lesion site. **(C)** Long term (weeks-months) tissue reaction. A glial scar forms around the implant, insulating it from surrounding healthy tissue. Neuronal degeneration/cell death may occur in the proximity of the device.

FBR is initiated by the neurovascular and cortical damage caused by probe insertion, which severs axons and neuronal cell bodies and disrupts the blood brain barrier (BBB) (Figure 3A).

By 24 h after device implantation, astroglia and microglia start to proliferate and migrate toward the injury site (Figure 3B), progressively forming a compact cellular sheath around the device (Figure 3C), thereby increasing the recording impedance and the distance between the microelectrode and neurons (Saxena et al., 2013; Kozai et al., 2015; Ereifej et al., 2018).

Moreover, the release of pro-inflammatory factors (such as IL-1, IL-6, and TNF-α) from macrophages, activated microglia and astroglia at the probe-tissue interface, together with enhanced levels of Reactive Oxygen Species (ROS) and unfavorable mechanical cues, create a hostile microenvironment for neurons and ultimately lead to neuronal cell death (Wellman et al., 2019). This, in turn, further increases the distance between neurons and microelectrodes, leading to loss of recording performances of neuronal spiking activity when this distance is greater than 140–150 μm (Henze et al., 2000; Holmgren et al., 2003). In the following paragraphs, the main components of the FBR process will be extensively detailed.

#### 2.3.1. Neurovascular and cortical damage

The neurovascular and cortical damages caused by the implantation of a neural probe constitutes the initial trigger of FBR.

The disruption of the BBB allows the recruitment of serum protein, blood borne macrophages, erythrocytes, activated platelets and clotting factors to the site of the injury. In addition, the BBB breach leads to the infiltration of neurotoxic factors, proinflammatory myeloid cells and ROS, consequently leading to oxidative stress and excitotoxicity in the local tissue around the implant. Moreover, ROS directly downregulate proteins responsible for tight junctions, establishing a positive feedback loop with the BBB disruption (Abdul-Muneer et al., 2015). The accumulation of fluid and necrotic nervous tissue around the implanted device also causes edema and an increased local pressure (Polikov et al., 2005; Bennett et al., 2018). Following BBB disruption, components of the complement cascade have been found at enhanced concentrations in the injured brain region, potentially contributing to the generation of a local innate immune response (Bennett et al., 2021).

In the acute phase of implantation, plasma proteins that extravasate due to BBB rupture/leakiness are adsorbed on the surface of the probe, forming a provisional matrix. The Vroman effect describes the continuous process of plasma protein adsorption and desorption from the surface of the probe which leads to the formation of this provisional matrix (Hirsh et al., 2013). High mobility proteins, such as albumin, are adsorbed at first and are gradually replaced by less motile proteins with a higher specific affinity for the probe's surface, such as fibrinogen, fibronectin, and vitronectin (as schematized in Figure 3A) (Klopfleisch and Jung, 2017). Surface physicochemical properties of the probe, such as wettability, topography, chemical composition, and charge are important properties at this stage, because they determine the thermodynamic equilibrium of protein/probe interactions. The provisional matrix formed on the surface of the implant is considered to have a critical impact on the outcome of FBR, by influencing the severity of long-term inflammation and of the immune response.

In addition to the initial physical disruption of the BBB caused by the probe implantation, several studies have shown that the presence of an external body alone elicits chronic (up to 4 weeks) homogeneous leakage of macromolecules with a size up to 10 nm, which is not observed following stab wound insults where the foreign object is not left inserted. Such studies were based on the immunohistochemical detection of plasma proteins, such as Mouse Serum Albumin (Tian et al., 2011) and Mouse IgG (Winslow and Tresco, 2010; Potter et al., 2012).

Fluorescent polymer nanoparticles with a larger size (20 nm to 1 μm diameter) were also used to assess long-term BBB permeability in the presence of an implant (Sawyer and Kyriakides, 2013). Nanoparticles with diameters of 20, 200, and 500 nm were found in the brain parenchyma next to the implant up to 4 weeks post implantation (study duration), while 1 μm diameter particles were excluded. This indicates that the presence of a foreign body elicits large gaps in the BBB in a chronic setting.

#### 2.3.2. Glial encapsulation

Microglia and astrocytes are widely considered the two major cell types involved in the brain wound healing response and in FBR (Biran et al., 2005; Polikov et al., 2005; Seymour and Kipke, 2007).

Microglia primarily act as cytotoxic cells killing pathogenic organisms or as phagocytes secreting proteolytic enzymes to degrade cellular debris and damaged matrix. When blood vessels are severed or in the presence of a leaky BBB, microglia are indistinguishable from blood borne, monocyte-derived macrophages (Ajami et al., 2011).

Microglia exist in a highly branched state until they are activated via injury-mediated mechanisms. Upon activation, they begin to proliferate, assume a more compact morphology and upregulate the production of lytic enzymes to aid foreign body degradation. Following probe insertion, during the first 30–45 min, nearby microglia immediately extend processes toward the probe, ensheath the device (Sharon et al., 2021) and 6 h following probe insertion, microglia (<130 μm distance from probe surface) exhibit morphological characteristics of a transitional stage to a reactive state (Kozai et al., 2012a; Wellman and Kozai, 2017). Recently, innate immunity activation pathways of microglia/macrophages have been investigated in the context of FBR. In particular, multiple studies have looked into the role of the innate immune receptor cluster of differentiation 14 (CD14), a molecule primarily expressed on microglia and circulating monocytes and associated with the recognition of pathogen associated and damage associated molecular patterns. The complete inhibition of CD14 resulted in an acute (2 weeks) but not chronic (16 weeks) improvement of histological endpoints (Bedell et al., 2018b), while a more selective suppression of CD14 only in blood derived cells yielded a longer lasting improvement of device electrical interfacing performances (Bedell et al., 2018a; Hermann et al., 2018b). These results, although not conclusive, may indicate that complete removal of CD14 is beneficial at acute time ranges, whereas limited CD14 signaling (from brain resident microglia) is beneficial at chronic time ranges. The impact of two additional receptors tightly associated with CD14 activation, Toll-like receptors 2 and 4, has also been investigated by Hermann et al. (2018a), but in this case the authors did not observe any beneficial effect from the partial or complete inhibition of these molecules. Recently, Franklin et al. (2023) have assessed the role of inflammasomes in the innate immune response to implanted neural devices.

Astrocytes constitute another key cellular component of FBR. While they were initially assumed to serve as little more than passive physical support elements for neurons, it is now clear that they play a key role in healthy physiology, in brain development and in the pathology of the nervous system (Kimelberg and Norenberg, 1989; Sofroniew and Vinters, 2010).

In the presence of various types of brain insults, and potentially through different activation pathways, astrocytes undergo numerous cytological, histochemical and biochemical changes, including increases in nuclear diameter, elevated DNA levels, accumulation of intermediate filaments, elevated oxidoreductive enzyme activity and increased synthesis of GFAP (Bovolenta et al., 1992). Astroglial swelling is also reported among the first responses in the presence of brain injury. It is likely that chemical factors released by injured neurons such as potassium, glutamate and lactate are responsible for astrocytic swelling. BBB disruption may also induce astrocytes to swell by taking up excess protein and water released in the extracellular environment (Michael, 1994).

Astrocytes and microglia initially form a layered sheet that insulates the foreign body from surrounding healthy brain tissue. By approximately 4–6 weeks, they begin to form a dense scar around the device that can last for years (Salatino et al., 2017).

Besides insulating the probe and physically displacing neurons from the electrode's proximity, activated astrocytes and microglia release proinflammatory cytokines, that induce excitotoxicity and neurodegeneration.

Interestingly, a recent ultra structural study reported a remarkable tissue re-generation around and in contact with a flexible polyimide implant was found after 8 weeks, which led to a recovery of neuronal cell body densities at a distance of ~ 1 μm from the microelectrodes surfaces (Sharon et al., 2021).

In addition to microglia and astrocytes, oligodendrocyte precursor cells, also referred to as NG2-Glia, have been shown to become activated and migrate toward the injury site (Wellman and Kozai, 2018). The delayed time-course of their activation (days) may indicate that they play a role in the formation of the outer layers of the glial scar.

#### 2.3.3. Traditional biomarkers of FBR

Post-mortem immunohistochemistry/immunofluorescence are still the most commonly used methods to assess the extent of FBR in the presence of intracortical neural probes. These strategies generally employ primary antibodies to assess the distribution of relevant antigens in the proximity of an implanted device and secondary antibodies, with specific affinity for the primary ones, to visualize it.

Table 1 shows the biomarker antigens that are most frequently targeted with these techniques, based on the category of FBR component that they represent. Despite being relatively easy to implement and allowing the determination of biomarker distribution with an extremely high spatial resolution, these strategies provide a relatively narrow view of the complex biological phenomena involved in FBR. In fact, their application requires an a priori selection of a relatively small subset of biomarkers of interest, typically selected among the ones presented in Table 1. To overcome this limitation and to identify novel FBR biomarkers and potential druggable targets, the application of transcriptomics to FBR assessment has recently emerged, as reviewed in the following paragraph.

**Table 1:** Biomarkers used for the immunohistochemical assessment of FBR.

**FBR component**	**Biomarker name**	**Description**
Glia/Myeloid cells reactivity	GFAP (Biran et al., 2005; Saxena et al., 2013; Wellman et al., 2019)	Glial fibrillary acidic protein (GFAP) is an intermediate filament protein which is overexpressed in reactive astrocytes and is commonly used to assess the extent of gliosis in the presence of a foreign body
	IBA-1 (Kozai et al., 2012b; Nguyen et al., 2014; Wellman et al., 2019)	Ionized calcium binding adapter molecule 1 (IBA1) is a protein involved in motility-associated rearrangement of the cytoskeleton overexpressed by microglia
	OLIG2 (Wellman et al., 2019)	OLIG2 is an oligodendrocyte specific transcription factor used as a marker for oligodendrocytes
	NG2 (Wellman et al., 2019)	The nerve/glial antigen 2 (NG2) is a chondroitin sulfate proteoglycan which is used as a marker for oligodendrocyte progenitor cells in the CNS
	Tryptase (Saxena et al., 2013)	Tryptase is a neutral protease concentrated in the secretory granules of mast cells, which serves as a marker of mast-cell activation
	Ninjurin (Saxena et al., 2013)	Ninjurin is a protein expressed on monocytes which mediates the transmigration of peripheral blood cells into the central nervous system
	CD68 (Biran et al., 2005; Nguyen et al., 2014; Wellman et al., 2019)	CD68 is a glycosylated type I membrane protein predominantly expressed in late endosomes and lysosomes of macrophages. CD68 is widely used as a pan-macrophage marker
	CD14 (Saxena et al., 2013)	CD14 is a glycosylphosphatidyl-inositol-anchored protein that functions as an innate immune receptor
	CD4 (Saxena et al., 2013)	CD4 is a monomeric type I transmembrane glycoprotein used as a T cell marker
	CD8a (Saxena et al., 2013)	CD8 is a cell surface glycoprotein found on most cytotoxic T lymphocytes
	CD32 (Saxena et al., 2013)	CD32 is a surface receptor glycoprotein expressed by a variety of immune cells. In the work of Saxena et al. it was used to assess the distribution of macrophages
	CD86 (Saxena et al., 2013)	CD86 is a glycoprotein constitutively expressed on dendritic cells, Langerhans cells, memory B cells, germinal center B cells, and macrophages. In the work of Saxena et al. it was used to assess the distribution of macrophages
Neuronal viability	NeuN (Biran et al., 2005; Saxena et al., 2013; Wellman et al., 2019)	NeuN is a Neuronal nuclear protein expressed by most type of neurons in the nervous system. It is commonly used to assess neuronal viability
	NF (160 or 200) (Biran et al., 2005; Winslow and Tresco, 2010; Wellman et al., 2019)	Neurofilament (NF) proteins are neuron-specific type IV intermediate filaments constituting a structural element of axons and synapses
	MAP-2 (McConnell et al., 2009; Winslow and Tresco, 2010; Winslow et al., 2010)	Microtubule associated protein-2 (MAP-2) is a protein involved in microtubule assembly. It is used as a marker for dendrites and for synaptic plasticity
	MBP (Winslow and Tresco, 2010)	Myelin basic protein (MBP) is a structural protein that plays a role in the organization of myelin sheaths of oligodendrocytes and Schwann cells. It is often considered a marker of active demyelination
	Phosphorylated Tau (McConnell et al., 2009; Saxena et al., 2013)	Phosphorylated Tau protein is indicative of neurodegeneration. AT8, pT231 and anti-PHF1 are antibodies used to recognize different categories of phosphorylated Tau
	Caspase-3 (Wellman et al., 2019)	Caspases are a family of cysteinyl aspartate-specific proteases that function as central regulators of apoptosis. Caspase-3, has been identified as a key mediator of apoptosis in neuronal cells
BBB breach	EBA (Kozai et al., 2012b)	Endothelial barrier antigen (EBA) is a membrane protein expressed by endothelial cells of the rat BBB, which has been shown to inversely correlate with the extent of BBB breach and leakage
	IgG (Winslow and Tresco, 2010; Saxena et al., 2013; Wellman et al., 2019)	Immunoglobulins G (IgG) are a class of antibodies constituting one of the main components of humoral immunity. In physiological conditions they are secluded in the vascular domain of the brain, but in case of BBB breach they diffuse in the parenchymal domain
	Albumin (Saxena et al., 2013)	Albumin is a globular serum protein that constitutes around 50 % of the proteins in plasma, which is secluded in the vascular domain of healthy brain tissue. However, similarly to IgG, its extravasation is reported in the case of BBB breach
	PDGFR-β (Wellman et al., 2019)	Platelet derived growth factor beta (PDGFR-β) is used as a marker of pericytes
	MMP (Tian and Kyriakides, 2009)	Matrix metalloproteinases (MMP) are enzymes that have been shown to degrade components of the basal lamina and disrupt the BBB

#### 2.3.4. Transcriptomics applied to the investigation of FBR

In recent years, molecular biology methods have been employed to achieve a broader understanding of the complex biological phenomena underlying FBR. In this context, transcriptomics emerges as a key tool to allow to simultaneous assessment of the expression of hundreds to thousands of genes, obviating the need to pre-select a limited number of biomarkers of interest, which is a requirement for immunohistochemical/immunofluorescence and qPCR-based strategies. Table 2 summarizes the strategies and outcomes of studies investigating FBR through transcriptomics.

**Table 2:** Summary table of the studies applying transcriptomics to FBR assessment.

**Study**	**Transcriptomics method**	**Number of assessed genes**	**Time points**	**Spatial resolution (or radius of collected tissue sample)**	**Number of DE genes**
Thompson et al. (2021)	RNA-Seq	Whole transcriptome	24 h, 1, and 6 weeks	Interfacial sample: 100 μm radius Distal sample: 500 μm radius	Interfacial vs. naïve: 157 Interfacial vs. distal: 94 Distal vs. naïve: 21 (across all time-points)
Joseph et al. (2021)	Clariom S Array from ThermoFisher	>20,000	4-h, 1, 2, 4, and 18 weeks	1 mm radius	14 (18 weeks time-point)
Bedell et al. (2020)	nCounter Mouse Neuroinflammation Plus panel RNA microarray from NanoString	777	6-h, 24-h, 72-h, and 2 weeks	250 μm radius	All 101 genes related to innate immune reaction (only ones analyzed in this study) were up regulated
Song et al. (2022)	nCounter Mouse Neuroinflammation Plus panel RNA microarray from NanoString	791	6-h, 24-h, 72-h, and 2 weeks	500 μm radius	419 (across all time-points)
Whitsitt et al. (2021)	Spatial transcriptomics	Whole transcriptome	24-h, 1, and 6 weeks	55 μm diameter spots	5,811 (across all time points)

The most general approach to assessing the local change in gene expression induced by invasive neural probes, is to perform RNA sequencing (RNA-seq) on tissue samples collected at different distances from the implant site. Thompson et al. (2021) applied this methodology on interfacial (100 μm from implant site) and distal (500 μm from implant site) brain tissue samples and found significant differential expression (DE) both among these samples and in their comparison with naïve brain tissue. Besides identifying genes traditionally associated with FBR, the authors were also able to point out novel potential mechanisms associated with neuronal, oligodendrocytic, microglial, and astroglial function. The following year, the same research group also presented a computational study aimed at extending the impact of their observations (Moore et al., 2022). They performed a differential co-expression analysis on their RNA-Seq dataset to identify coordinated expression of genes, which could implicate the activation of specific regulatory pathways and allow the identification of novel hub gene targets for FBR reduction. Hub genes associated with cellular structure, synaptic plasticity, axonal transport, and metabolism were identified through this approach, although direct validation is still missing.

Other research groups have employed a different approach to analogous transcriptomics studies. Rather than performing RNA-seq to assess DE in the whole genome, mRNA extracted from tissue is hybridized with a pre-set of capture and reporter probes, available through commercial transcriptomic microarrays. Some of these RNA hybridization kits are very broad, such as the one used by Joseph et al. (2021) encompassing more than 20,000 genes, while others focus on a limited number of genes mainly associated with neuroinflammation, such as the nCounter^®^ Mouse Neuroinflammation Panel by NanoString Technologies, used in the works of Bedell et al. (2020) and Song et al. (2022). Although this kit provides information on the expression of a relatively large number of genes (770), it may limit the chances of spotting alternative regulatory pathways of FBR, not strictly related to neuroinflammation.

Joseph et al. (2021) performed the longest (18 weeks) transcriptomics study to date, assessing long-term brain tissue responses to flexible, minimally tethered probes. The authors observed a transcriptional profile of implanted animals similar to that of non-implanted controls, with an increased expression of genes associated with wound healing and angiogenesis, which is consistent with the probe design and tethering strategy they adopted. However, a significant enrichment of genes related to gliogenesis and glial cell differentiation was reported after 18 weeks, consistent with the results of immuno-fluorescence imaging. The authors also observed an elevated expression of genes belonging to the family of intermediate early genes, which may arisen due to the continuous interfacial stress caused by brain micromotion.

Although the application of transcriptomics to the study of FBR paves the way to a deeper understanding of its underlying mechanisms of action, this strategy also presents drawbacks and potential limitations. The main technical pitfalls are that the generation of datasets is resource intensive and that their interpretation can be difficult. Two conceptual limitations of this strategy are that gene expression does not always align with protein expression and that this method does not elucidate whether DE is driven by altered phenotypes at a cellular level or by changes in the overall cellular population. To address these issues and to validate mRNA as a predictor of protein expression, Thompson et al. (2023) performed an immunohistochemical analysis to evaluate the distribution of a sub-set of proteins identified through a previous RNA-Seq analysis (Thompson et al., 2021). In this work, the authors observed that all the proteins identified through transcriptomics and quantified with immunofluorescence were in some way disrupted, although the expression of some of them did not match the observations from RNA-seq. This result suggests that although protein expression does not always reflect gene expression, it is possible to use RNA-seq to predict broad and cell-type specific changes of proteins involved in FBR.

A further limitation to the use of a standard transcriptomics approach for FBR assessment is that it does not provide spatial resolution. Typically, a relatively large portion of brain tissue around the probe (diameter in the range of hundreds of μm) is extracted from brain tissue slices and pooled to assess DE compared to naïve brain tissue. A strategy to overcome this limitation is the application of recently introduced spatial transcriptomics (Ståhl et al., 2016), which allows a spatial resolution in the range of 10–15 cells per spot (50–100 μm diameter spots). Whitsitt et al. applied this strategy, in combination with immuno fluorescence, both for FBR assessment (Whitsitt et al., 2021) and to understand the impact of electrical stimulation on the genetic expression of surrounding brain tissue (Whitsitt et al., 2022).

Overall, transcriptomics appears to be a promising avenue to achieve a deeper understanding of FBR and to identify novel biomarkers of interest and druggable targets to improve the integration of neural probes within brain tissue, particularly when it provides high resolution spatial information. The recent introduction of high-definition spatial transcriptomics (Vickovic et al., 2019) appears to be a key step toward this direction, although it has thus far never been implemented in the context of FBR assessment. However, to this date, immunofluorescence or immunohistochemistry are still required to validate the results of transcriptomic studies and appears to be a more practical approach for FBR assessment.

## 3. Factors influencing chronic probe-tissue interactions

Multiple factors have been shown to influence the way neural probes interact/integrate within brain tissue in a chronic setting.

**Figure 4:**
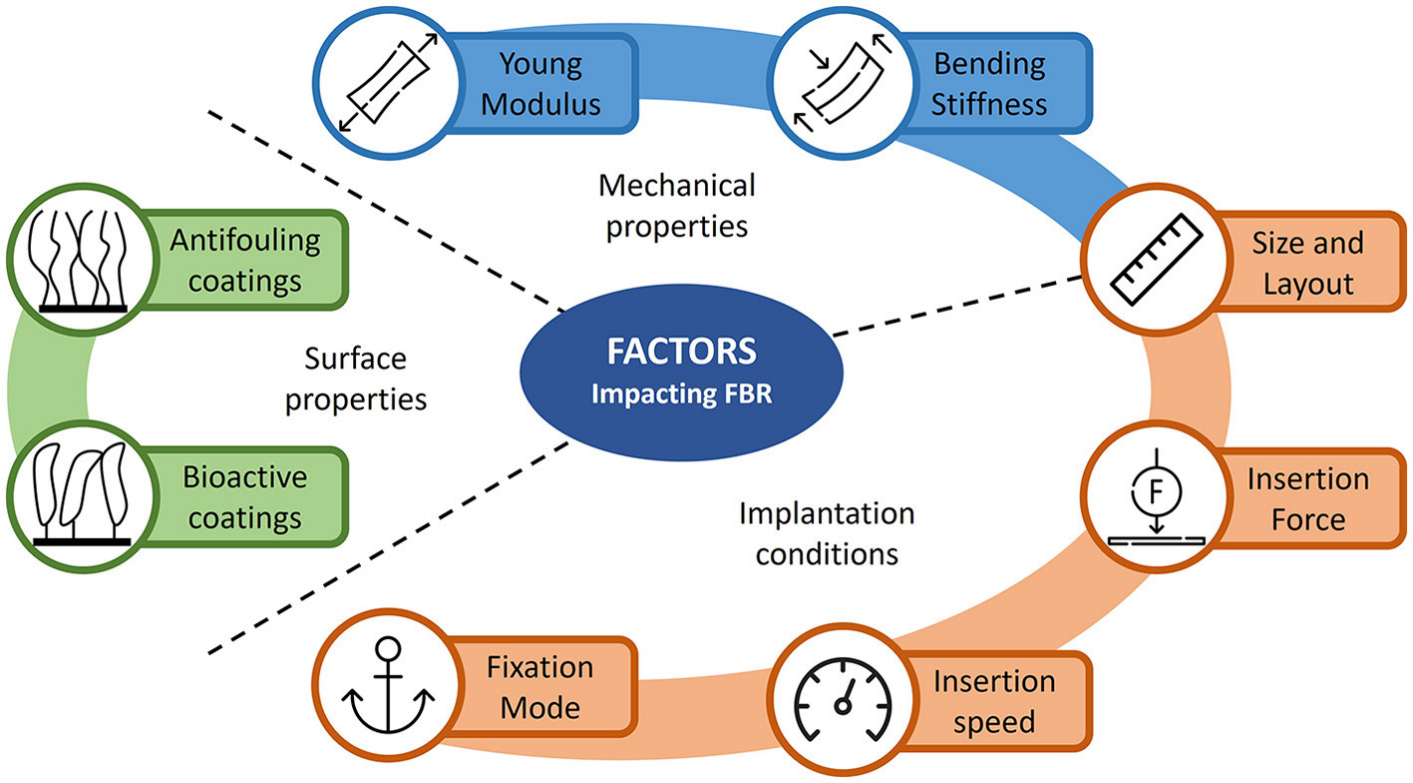
Illustration of the different FBR-related factors influencing the stability of chronic neural implants. The various factors are divided in three main categories: mechanical and surface properties of the neural probe and implantation conditions.

Some of these factors are related to the design of the probe, such as size and layout, mechanical and surface physicochemical properties of the device. Other factors are related to the implantation procedure. Figure 4 summarizes some of these factors and their impact on FBR is reviewed in the following paragraphs.

### 3.1. Probe mechanical properties

The Young's modulus of the substrates used to fabricate neural probes is typically 6 to 8 orders of magnitude larger than the one of brain tissue. Polymeric materials such as Parylene, Polyimide, and SU-8 have a Young's modulus in the order of a few GPa, while stiffer materials such as silicon and metals have a Young's modulus in the order of hundreds of GPa.

The mismatch in mechanical properties between brain tissue and neural probes is considered one of the key factors leading to sustained FBR to implanted neural probes, particularly in a chronic setting where the impact is amplified by the constant micromotion of brain tissue around the implanted device.

Besides the bulk Young's modulus of the substrate material of the probe, an important mechanical parameter that determines the probe-tissue interaction is their bending stiffness.

Based on the theory of solid mechanics, the force applied to a cantilever beam and the displacement that it induces are related by the following equation:


(1) $$\begin{array}{l} \delta =\frac{Fl^{3}}{3EI} \end{array}$$

Where δ is the displacement induced, F is the applied load, l is the length of the beam, E is the Young's modulus of the substrate and I is the area moment of inertia of the cross section.

The bending stiffness is the coefficient (K) that linearly correlates the force applied to a cantilever beam (F) and the displacement it induces (δ):


(2) $$\begin{array}{l} F=K\delta \end{array}$$

Therefore, the bending stiffness K can be derived from Equations (1) and (2) as:


(3) $$\begin{array}{l} K=\frac{3EI}{l^{3}} \end{array}$$

**Figure 5:**
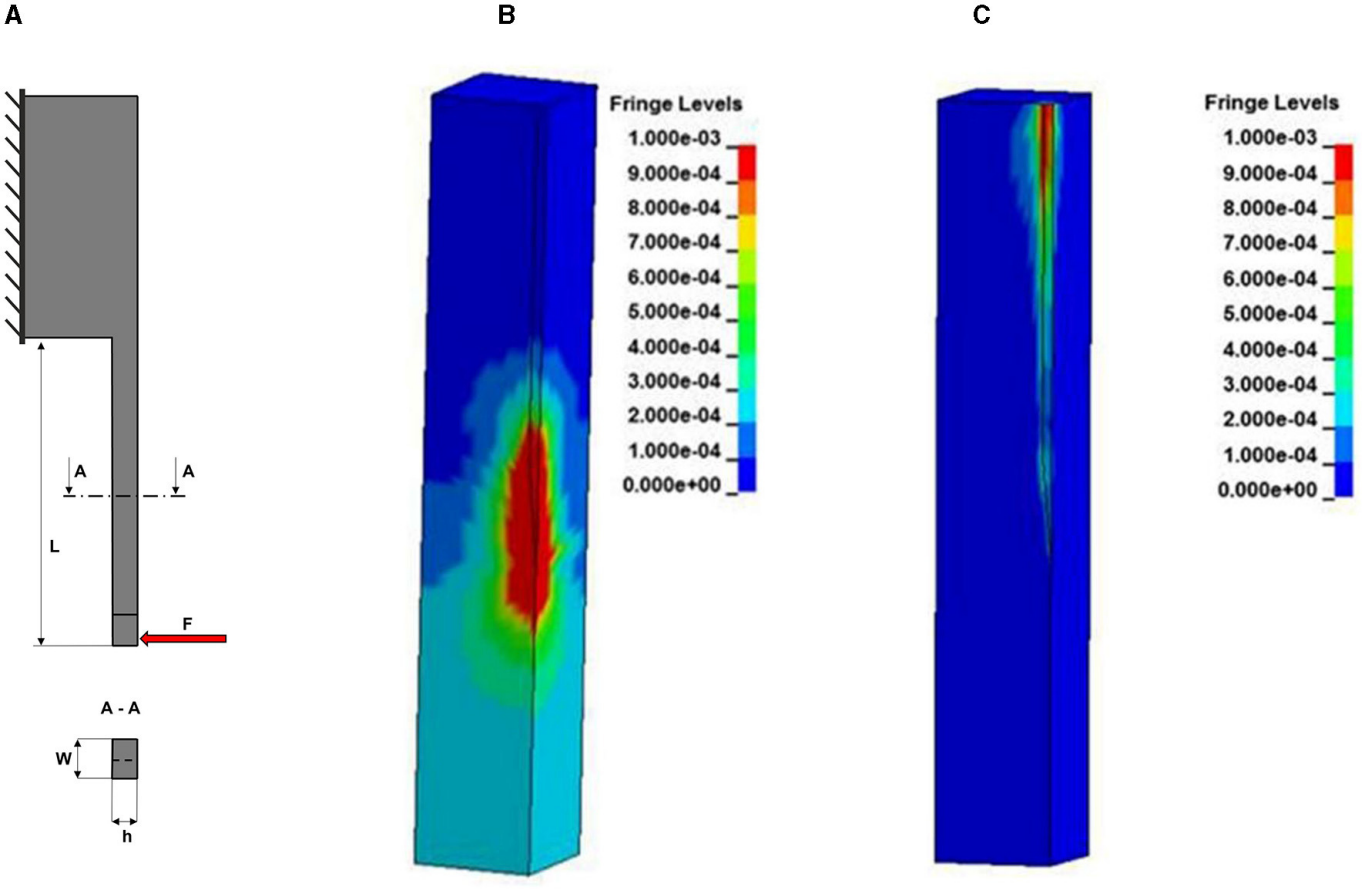
Simulations of relevant mechanical properties of implantable probes. **(A)** Scheme of a probe illustrating the geometrical parameters that contribute to its bending stiffness. The geometry of the probe is modeled as a cantilever beam with a constant cross section fixed at one end (probe base) and free to move at the opposite end (probe tip) where a force F can be applied to estimate different properties. **(B)** Simulated strain distribution in the brain with 4 μm micromotion amplitude for a silicon probe. **(C)** Simulated strain for a probe with a 200 kPa stiffness modulus. **(B, C)** Are adapted from Polanco et al. (2016).

The bending stiffness is proportional to the Young's modulus of the substrate (E) but also to the area moment of inertia of the beam's cross section (I). The moment of inertia is inversely proportional to the cube of the beam length (L). For a rectangular cross-section, the moment of inertia is given by:


(4) $$\begin{array}{l} I=\frac{WH^{3}}{12} \end{array}$$

while for a circular cross-section it is given by:


(5) $$\begin{array}{l} I=\frac{\pi d^{4}}{64} \end{array}$$

where W and H are respectively the width and the height of the rectangular cross section and d is the diameter of the circular cross-section. All these geometrical parameters of the probe that are relevant to FBR are illustrated in Figure 5A.

#### 3.1.1. Probe Young's modulus

The impact of substrate stiffness on mechanical stress at the biotic-abiotic interface has been estimated through finite element method (FEM) models (Subbaroyan et al., 2005; Polanco et al., 2014, 2016). Based on two of such models (Subbaroyan et al., 2005; Polanco et al., 2014), the stiffness discrepancy between neural probes and brain tissue must be lower than 3 orders of magnitude to significantly reduce the micromotion stress. Therefore, substrates such as Polyimide do not seem to provide a substantial mechanical advantage compared to silicon in applications involving longitudinal loading (Polanco et al., 2014). Figures 5B, C represents the difference in simulated strain distribution for probes with different Young's moduli. The impact of bulk mechanical properties on tissue reaction was also assessed. Nguyen et al. (2014) compared the tissue reaction to mechanically adaptive probes and to stiff silicon probes. Although they observed a similar acute tissue response, they reported an improved neuroinflammatory response at later time-points (of more than 2 weeks).

In another study (Harris et al., 2011), the same probes were compared to stiff metal microwires (160 μm overall diameter). The glial scar response to compliant probes was less vigorous than to stiff wires; however, in this case long-term (8 weeks time point) neuronal survival was comparable between the two types of devices. This outcome might be explained by the different geometry of the two probes, which may lead to different iatrogenic injury and to a different distribution of inflammatory and cytotoxic molecules at the probe-tissue interface.

An important observation made by Lee et al. (2017) is that there may be a limit to the relevance of the probe Young's modulus for FBR reduction. In fact, the authors reported a significant reduction of FBR biomarkers for polymeric probes compared to silicon probes, but no consistent differences among the different “flexible” probes, despite a difference in Young's modulus spanning several orders of magnitude.

Overall, the Young's modulus of materials constituting both the surface and the bulk of neural probes seems to play an important, but not exclusive role in determining the extent of FBR (Stiller et al., 2018).

#### 3.1.2. Bending stiffness

A meta-analysis performed on the data from nine studies demonstrated that the severity of the immune response is highly correlated with the bending stiffness of the device, as opposed to the bulk Young's modulus or to the cross-sectional area independently (Stiller et al., 2018).

Some research groups aim to achieve a disruptive reduction in device bending stiffness by acting both on the Young's modulus and on cross-sectional dimensions. This approach has been shown to produce favorable results, both based on immunohistochemical analysis and based on the probe's ability to stably track single unit activity for weeks or months.

Luan et al. (2017) fabricated flexible nanoelectronic thread (NET) probes with subcellular cross-sectional dimensions, as small as 10 by 1.5 μm^2^. This allowed to reduce the overall bending stiffness of the device by several orders of magnitude compared to typical neural probes. This approach, which was also recently followed by Zhao et al. (2022) led to a seamless integration of probes within brain tissue and the reliable tracking and detection of single unit activity for months.

Another approach to achieve brain-like ultraflexibility is to generate macroporous mesh structures with feature sizes comparable to neuronal soma (Liu et al., 2015; Xie et al., 2015; Fu et al., 2016; Zhou et al., 2017). While this solution allows to readily scale the number of recording sites, the use of a syringe for implantation induces higher insertion trauma and bleeding, with a potential negative impact on both acute and chronic performances.

A noteworthy effort in the direction of reducing the bending stiffness was reported by Yang et al. (2019). In this case, they designed and fabricated bio-inspired neuron-like electronic devices with a unit building block that is structurally and mechanically similar to a neuron, reaching a cross-sectional dimension of the neurite-like interconnect portion of the device as low as 2 μm wide and 0.9 μm thick. The interpenetration of neuron-like electronics with biological neurons is similar to that of natural brain tissue and the authors were able to track stable single unit activity for the 3 months duration of the study.

The use of neural probes with a ultra low bending stiffness, however, also poses constraints, particularly during the surgical implantation procedure. Recently, a series of methods to aid the implantation of highly compliant neural probes have been reviewed (Thielen and Meng, 2021).

### 3.2. Probe size and layout

**Figure 6:**
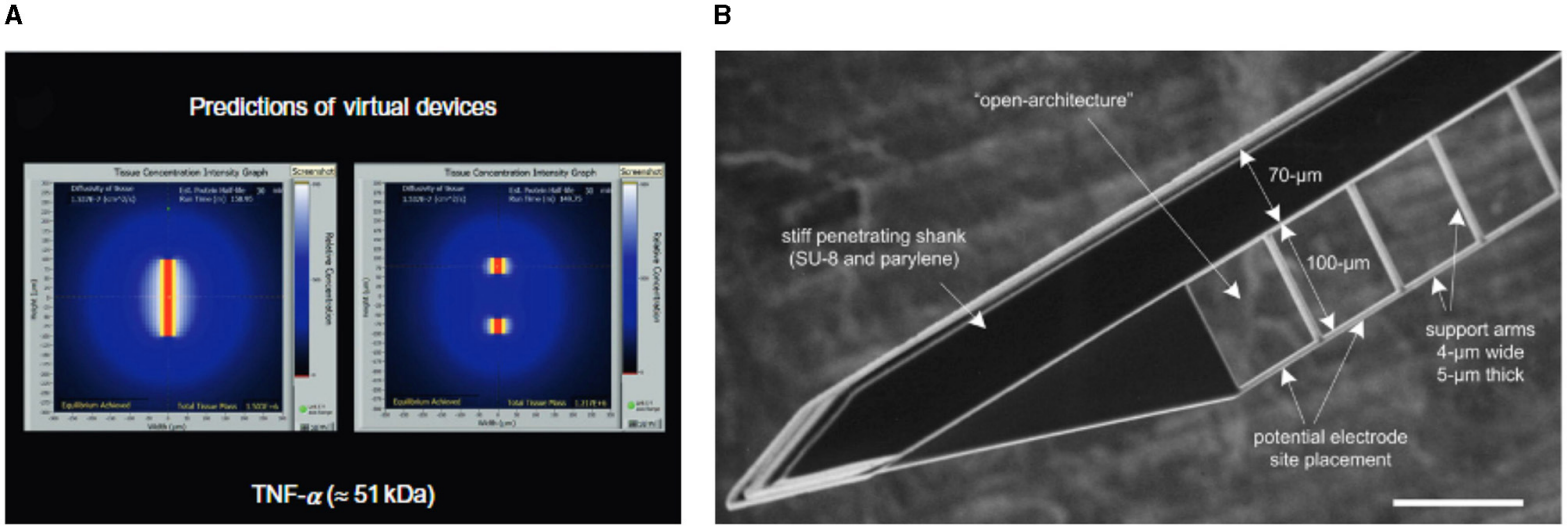
Simulations and devices to study the impact of probe sizes and geometries on FBR. **(A)** Simulated inflammatory factors (TNF-α) surrounding the cross section of solid (left) and lattice (right) devices with similar penetrating profiles but different exposed surface area. Adapted from Skousen et al. (2011). **(B)** SEM micrograph of the thin adjoining lateral structure developed by Seymour and Kipke to study the impact of size on tissue reaction. Scale bar 100 μm. Adapted from Seymour and Kipke (2007).

The size and shape of cortex penetrating neural probes are other important factors that play a role in determining the extent of FBR (Gori et al., 2021):

- A reduction of probe size entails a lower bending stiffness of the neural probe, which will result in lower micromotion-induced stress (Stiller et al., 2018)
- Neural probes with a smaller footprint displace/injure a smaller volume of brain tissue and cause less extensive BBB disruption and cortical damage (Obaid et al., 2020b)
- Smaller probes expose less surface area to biological tissues, reducing the interfacial accumulation of inflammatory and cytotoxic molecules, as shown in Figure 6A (Skousen et al., 2011)

Stice et al. (2007) implanted insulated stainless steel wires, with a diameter of 12 and 25 μm, to assess the influence of size on FBR. The 12 μm wires were coated with Polyglycolic acid (PGA) in order to avoid buckling. The authors observed no difference in the initial tissue response (first 2 weeks), which may be explained by the similar cross sectional area during implantation, due to the PGA layer on the thinner microwire. However, 4 weeks after implantation, glial scarring around the implant site was significantly reduced for the 12 μm wire.

Further evidence toward this conclusion is provided by Thelin et al. (2011) and Spencer et al. (2017), who observed that increasing the size of cylindrical probes increased glial scarring, local BBB permeability and macrophage activation, while decreasing local neuronal density (Spencer et al., 2017).

Skousen et al. (2011) studied chronic FBR to solid silicon probes and lattice arrays with identical penetrating profiles but with reduced surface area. Presenting less surface area led to less persistent macrophage activation, decreased BBB leakiness and reduced neuronal cell loss. The reduced surface area and the increased clearance of soluble factors were considered the primary factors for FBR reduction.

Similar observations were made by Seymour and Kipke (2007), who studied tissue reaction around a thick parylene probe supporting a 5-μm-thick lateral platform (shown in Figure 6B). Non-neuronal density around the thin lateral structure was less than one third compared to the corresponding region of the shank, while neuronal density was about one-third higher. Overall, these results support the hypothesis that presenting reactive cells with a narrow edge (subcellular dimension) prevents attachment and spreading and induces a more favorable distribution of inflammatory molecules.

This observation is consistent with the ones made by Kozai et al. (2012a) and Luan et al. (2017) for neural probes with a subcellular cross-sectional dimension. In both cases, the authors reported an improved integration within brain tissue compared to larger devices. Therefore, neural probes with sub-cellular dimensions seem a promising path for a drastic reduction of FBR, although they pose constraints in terms of the area available for integration and interconnection of microelectrodes.

Finally, the positioning of electrode sites on the device seems to play an important role in the quality of chronic electrical interfacing. Electrodes closer to the edge of the device were reported to outperform those in the center (Fiáth et al., 2021). Kilias et al. (2021) exploited both size reduction and electrode placement by fabricating microelectrodes on thin polyimide wings attached to a stiff silicon backbone. This approach allowed to stably record electrophysiological activity for up to 104 days and outperformed electrodes placed directly on the stiff silicon backbone.

### 3.3. Surface physicochemical properties

Surface physicochemical properties have also been shown to play an important role in chronic interactions with the brain. Probes with a more compliant surface impose a significantly lower distribution of strain values compared to non-compliant probes (Sridharan et al., 2015).

Various strategies have been used to engineer the probe surface properties, most notably by integrating polymers or hydrogels, usually via dip coating or by covalent bonding (Azemi et al., 2011; Lecomte et al., 2018). Spencer et al. (2017) tested the ability of PEG hydrogel coatings to modulate glial scarring by matching the mechanical properties of the brain. However, the benefit of reducing mechanical mismatch at the probe-tissue interface needs to be carefully balanced with the increase in cross-sectional dimensions it requires.

**Figure 7:**
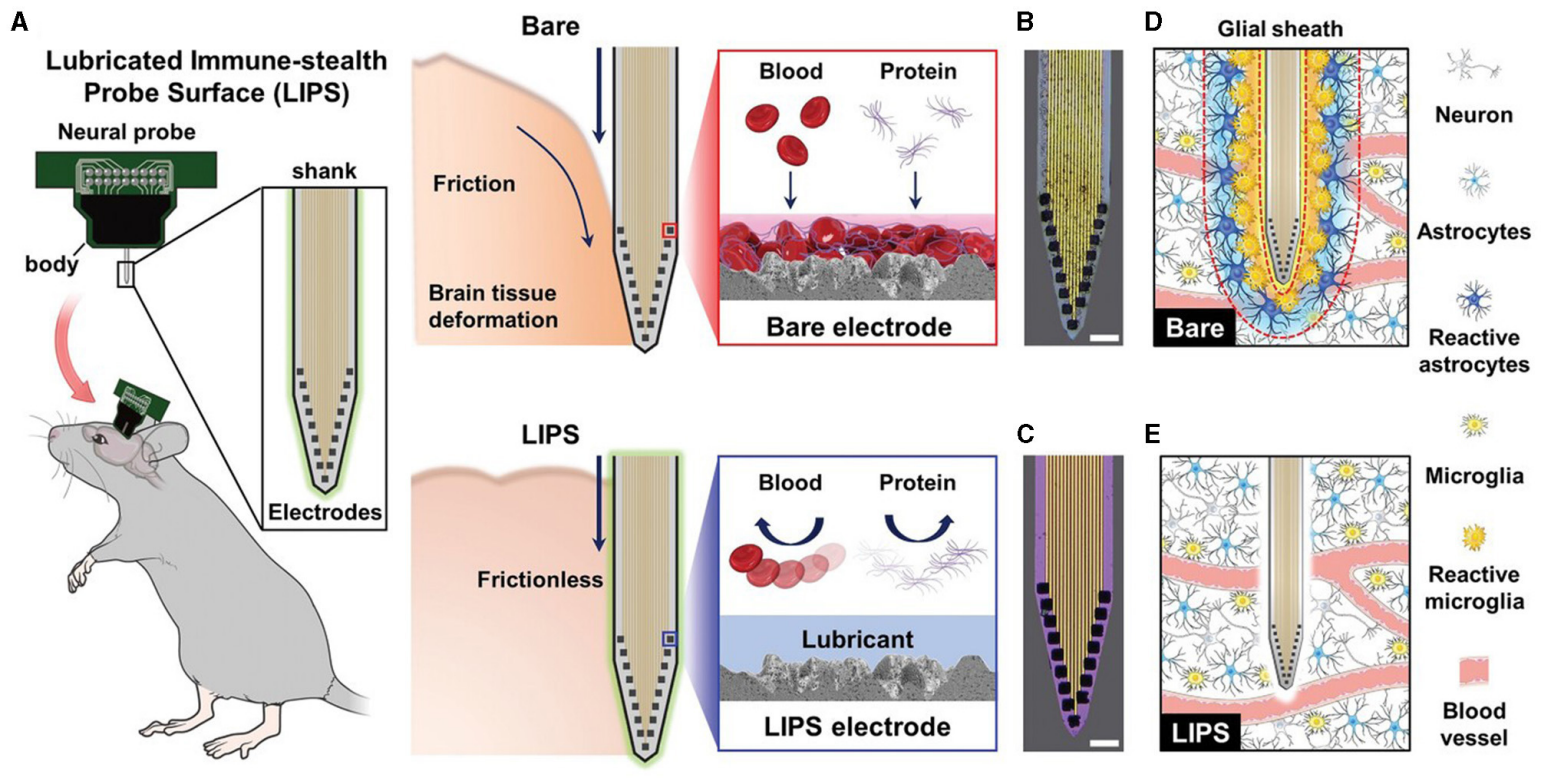
Optimization of surface properties to minimize tissue responses using the LIPS approach. **(A)** Schematic of the mechanism for minimizing the immune response exploited by LIPS-coated probes compared to bare probes. Optical microscope images of the **(B)** bare and **(C)** LIPS probes submerged in blood for 30 min and retrieved, displaying the antifouling properties of LIPS coating. Scale bars, 50 μm. Schematic of glial encapsulation on the **(D)** bare and **(E)** LIPS probes. Adapted from Lee et al. (2021).

A different strategy is to lubricate neural probes to minimize friction during insertion. Lee et al. (2021) developed a lubricated immune-stealthy probe surface (LIPS), which was shown to significantly reduce insertion impulse in an agar gel and to prevent protein adsorption, improving the quality and longevity of neuronal signals (Figure 7).

Besides surface modification strategies acting on mechanical interactions between the device and brain tissue, other coating strategies aim to reduce FBR by preventing protein adsorption on the probe (antifouling coatings) or by presenting bioactive molecules at the probe-tissue interface (bioactive coatings).

#### 3.3.1. Antifouling surface modifications

The non-specific adsorption of serum protein on the surface of neural probes (biofouling), particularly fibrinogen, has been shown to drive the activation of microglia/macrophages (Hu et al., 2001).

An approach to target this issue is to coat neural probes with highly hydrophilic polymers (Lu et al., 2009; Kozai et al., 2012b; Rao et al., 2012; Gutowski et al., 2015) or with zwitterionic molecules (Golabchi et al., 2019; Zou et al., 2021).

In some cases, a covalent binding strategy was used, while in other cases a hydrophilic polymer was passively adsorbed on the surface of the device (Lu et al., 2009; Rao et al., 2012). This second approach generates much thicker layers (μm thickness) which dissolve over time, potentially leading to a loss in antifouling properties.

Antifouling strategies can also be combined with the delivery of bioactive molecules. For example, Gutowski et al. (2015) engineered a protease-degradable PEG antifouling coating which can release IL-Ra in response to tissue inflammation.

Coatings with both an antifouling as well as a bioactive function aiming to improve cell adhesion were also proposed, such as the EK-IKVAV modified electrodes presented by Zou et al. (2021). In this case, an EK zwitterionic peptide sequence head was used to generate a hydration layer with antifouling properties, while an IKVAV tail was used to increase the adhesion of neuronal cells to the microelectrodes. While there is no conclusive evidence that antifouling coatings alone may lead to an improved chronic performance, their combination with bioactive coatings and other strategies for FBR reduction appear promising.

#### 3.3.2. Bioactive coatings

Another strategy to improve biocompatibility of implanted neural probes is to deliver bioactive molecules/drugs at the biotic-abiotic interface or to functionalize their surface with molecules that modulate cellular responses.

The most commonly employed molecules for targeted delivery at the device-tissue interface are anti-inflammatory drugs such as the corticosteroid dexamethasone. Systemic administration of dexamethasone has been shown to affect early and sustained reactive responses to device implantation (Spataro et al., 2005); however, this delivery strategy is inefficient and may cause unintended side effects.

Several strategies can be employed for a more localized and efficient drug delivery:

- Convection enhanced drug delivery using microfluidic channels (Chen et al., 1997; Retterer et al., 2004; Frey et al., 2018; Wen et al., 2019).
- Custom-made drug-eluting coatings (Abidian et al., 2006; Kato et al., 2006; Zhong and Bellamkonda, 2007; Lee et al., 2013; Potter et al., 2014). Poly (vinyl alcohol), poly (ethylene oxide), PEG, nitrocellulose and matrigel are some of the materials used to fabricate drug-eluting coatings. Further control over release kinetics can be achieved by embedding drug-loaded nanoparticles in the coating (Kato et al., 2006; Mercanzini et al., 2010).
- Active drug-eluting materials (Boehler et al., 2017). These materials incorporate a drug (e.g., dexamethasone) in a conducting polymer coating (e.g., PEDOT-PSS). On-demand drug release is achieved by applying a cyclic voltammetry signal.

Besides dexamethasone, a drug that multiple studies have deemed as effective in reducing tissue reaction (Zhong and Bellamkonda, 2007; Mercanzini et al., 2008), other compounds have been proposed. The natural antioxidant curcumin was shown to initially improve neuronal survival and reduce BBB leakage over the first 4 weeks of implantation; however, this benefit was lost over a longer period (12 weeks) (Potter et al., 2014). Nerve growth factor (NGF) (Kato et al., 2006) and α-melanocyte stimulating hormone (α-MSH) (Zhong and Bellamkonda, 2005) have also been successfully loaded in the surface coatings of neural probes; however, their impact on FBR has not yet been assessed *in-vivo*.

A different strategy for a bioactive functionalization of the device is to bind protein/peptides to its surface, to improve neuronal adhesion and to decrease glial encapsulation. Azemi et al. (2008) covalently bound neuronal adhesion protein L1 to silicon neural probes. The authors observed an increased neuronal density and a decreased activation of astrocytes and macrophages around surface-modified probes. Oakes et al. (2018) implemented a non covalent bioactive surface modification by dip coating neural probes in astrocyte derived extracellular matrix (ECM) and observed a reduction in GFAP immunoreactivity at the 8-weeks timepoint, but no significant changes in neuronal density around ECM coated probes.

### 3.4. Implantation procedure

Most of the effort toward achieving seamless integration of neural probes within the brain focuses on design aspects influencing chronic interactions with the tissue, while acute damage caused by the implantation procedure is often overlooked. However, materials and components for the assembly of the device (or “device packaging”) can have a large impact on the implantation procedure, subsequent fixation to the skull, and therefore induce undesirable effects on the chronic implant.

In this context, the packaging of the device can influence the requirements for the implantation procedure and the subsequent fixation modality to the skull.

While different probe mounting strategies have been proposed, one approach to studying and potentially reducing tissue damage during implantation consists of measuring and controlling the insertion forces. The axial penetration force and tissue dimpling (the compression of brain tissue under the tip of the neural probe before actual penetration) may in fact be informative indicators of the degree of invasiveness of the implantation procedure and could be a valuable metric to optimize probe design and insertion procedure.

#### 3.4.1. Probe assembly and fixation mode

Neural implants can be rigidly tethered to the skull or decoupled from the skull using flexible interconnections to approach the conditions of a free-floating implant. Rigidly tethered probes have been reported to lead to oval-shaped cavities, with a cross-sectional area larger than the implant itself (Biran et al., 2007; Thelin et al., 2011) and significantly higher ED1 and GFAP expression, as well as decreased neuronal and axonal density (Biran et al., 2007). Indeed, implants rigidly tethered to the skull were reported to increase FBR compared to free floating implants (Kim et al., 2004; Biran et al., 2007; Thelin et al., 2011; Chauviere et al., 2019), most likely because this fixation modality increases the magnitude of the relative micromotion among the probe and the brain tissue.

Inertial forces resulting from the difference between the density of neural probes and the tissue have also been reported to increase FBR. Lind et al. (2013) tested glial scarring in the presence of untethered probes with similar size, shape, surface structure, and elastic modulus but with densities which differed by an order of magnitude. Under the tested conditions, low-density probes caused significantly smaller scars than high-density probes. This indicates that inertial forces can elicit substantial astrocytic reactions. By extension, this result indicates that forces arising from the micromotion at the tissue/implant interface may also have a significant impact on glial scarring.

#### 3.4.2. Insertion force

A few research groups studied the impact of parameters such as insertion speed (Sharp et al., 2009; Welkenhuysen et al., 2011; Fekete et al., 2015; Hosseini-Farid et al., 2019; Obaid et al., 2020b), probe size (Sharp et al., 2009; Hosseini-Farid et al., 2019; Obaid et al., 2020b), surface properties (Jensen et al., 2006; Sridharan et al., 2015), and tip shape (Fekete et al., 2015; Obaid et al., 2020b) on the magnitude of the insertion force of neural probes measured *in-vivo*.

Generally, an increase in insertion force with insertion speed is observed (Sharp et al., 2009; Fekete et al., 2015; Hosseini-Farid et al., 2019; Obaid et al., 2020b), which is consistent with the well-known viscoelastic properties of brain tissue. The extent of the reported increase in insertion forces depends both on tested insertion speeds, ranging from 2 μm/s to 1.7 mm/s in different studies, and on the size and layout of the tested probes.

The size of neural probe shanks, i.e., the part of the probe inserted in the tissue, is in fact another important predictor of the insertion force (Sharp et al., 2009; Welkenhuysen et al., 2011; Obaid et al., 2020b). This is due to the larger volume of displaced brain tissue induced by larger probes and to the wider area of the probe in contact with brain tissue, which yields higher frictional forces during insertion.

Interestingly, although only studied in a few works, the surface properties of neural probes have been reported to influence insertion dynamics and a reduced penetration force was reported for probes treated both with hydrophilic and hydrophobic coatings (Jensen et al., 2006).

Finally, the tip geometry has also been shown to influence insertion force. Electro-sharpened tungsten microwires exhibit remarkably different insertion dynamics compared to flat- and angle-polished microwires, particularly in terms of tissue dimpling (Obaid et al., 2020b). In addition, the tip angle of planar silicon probes was also shown to influence insertion force (Fekete et al., 2015).

Despite the information provided from force measurements, these studies rarely report post-implantation immunohistochemical analysis, which would allow to understand if a correlation exists between insertion force and acute tissue damage and chronic FBR. Most likely, this correlation exists and if demonstrated, insertion force measurements would constitute a valuable metric to rapidly validate insertion protocol and probe design, as well as assessing the execution of each probe implantation.

#### 3.4.3. Insertion speed

Another key parameter of the implantation procedure is the speed at which the probe is inserted into the tissue. However, no consensus regarding the impact of this parameter on tissue response has been reached so far. While some studies suggest that a slower penetration might result in less tissue damage (Fiáth et al., 2019), especially for smaller probes (Obaid et al., 2020b), others indicate that a faster insertion results in lower mean effective strain (Bjornsson et al., 2006). In this context, however, it is important to recognize that the definition of slow and fast insertions depends on the range of penetration speeds tested in the different studies, which spanned over seven orders of magnitude (from 1 μm/s to 10 m/s).

Mechanical (Rennaker et al., 2005) and pneumatic (Rousche and Normann, 1992) insertion devices have been proposed to implant neural probes with a speed higher than 1 m/s. The insertion of microwire electrode array with a velocity of 1.5 m/s through a mechanical inserter was shown to decrease tissue dimpling compared to a slower insertion (Rennaker et al., 2005). Moreover, the faster insertion procedure resulted in better chronic functional performances when recording neural activity. A potential explanation of the reported improved performance is the prevention of cortical tissue compression (referred to as tissue dimpling), which occurs as the tip of the probe presses on brain tissue, before rupturing its surface and beginning the actual insertion. Reduction of brain tissue dimpling during implantation may prevent traumatic brain injury (TBI) and ischemic damage. Rousche and Normann (1992) reported that a penetration speed of 8.3 m/s, achieved using a pneumatically actuated insertion system, allowed the complete penetration of the Utah Electrode Array. For slower insertion speeds, full penetration of the central electrodes could not be achieved.

In other works, neural probes were inserted at much slower velocities (below 2 mm/s). Bjornsson et al. (2006) observed that a faster insertion (2 mm/s) of sharp devices led to lower mean effective strain in *ex vivo* tissue compared to lower insertion speeds (125 and 500 μm/s).

However, Fiáth et al. (2019) observed that both the signal-to-noise ratio and the number of separable single units recorded were significantly higher for the slowest insertion speed (2 μm/s) compared to faster speeds (20, 100, and 1,000 μm/s). The different ranges of tested speeds in these two studies may explain their apparent discrepancies. In fact, the improvement in the recording quality reported by Fiath was significantly different only for the slowest insertion speed (2 μm/s), which is far below the experimental range tested by Bjornson.

## 4. Comparison between passive and active probes

MEMS technology has led to the development of passive implantable microelectrode arrays (either on silicon or polymer substrates) which allow to consistently record neuronal electrical signals from the brain. As previously reported, these passive probes rely on the individual routings of electrical interconnects between each electrode site and dedicated output pad. The latter is used for interconnection of the probe electrodes with external low-noise front-end amplifiers, either bulky instruments or, more recently, integrated front-end CMOS chips.

An alternative approach has emerged in the last 10 years which is based on the use of CMOS technology to create implantable active probes integrating an array of metal electrodes and the electronics required for signal conditioning and acquisition into the same silicon substrate.

### 4.1. Limitations of passive probes

Neural probes that do not implement active components, referred to as passive probes, are widely used but have major inherent limitations. Firstly, long routing lines between electrode sites and dedicated front-end neural amplifier lead to high-impedance nodes at the electrode sites which are sensitive to electromagnetic interference, signal attenuation due to parasitic capacitances, and crosstalk between adjacent channels. Secondly, despite impressive advancements in lithography, deposition, and micro/nano structuring processes, and despite the use of custom microfabrication techniques such as electron-beam lithography (Du et al., 2011), the number of individual routing traces that can be accommodated along the probe shaft remains a bottleneck for high channel count multi-electrode arrays.

In order to increase the number of recording sites on the probe and to decrease the resistance of routing lines, passive neural probes are typically produced with a tapered geometry, which allows more space for the integration of interconnecting metal lines. However, this increases the cross-sectional dimensions toward the base of the device, which comes with implications on FBR as described in the previous paragraphs.

### 4.2. Active CMOS technology and its advantages

The use of CMOS technology enables the circumvention of the above-mentioned limitations and the integration of high-channel count devices with dense and continuous electrode array arrangements. Firstly, this is obtained by placing an integrated circuit that converts the high-impedance node to a low-impedance node in close proximity to the recording site, which strongly mitigates coupling effects. Secondly, the integration of active circuitry on the probe enables the multiplexing of multiple electrode signals into a single output line or the selection of a subset of recordings sites (Fiáth et al., 2018). Due to these technological advancements, active CMOS probes do not require the typical tapered geometry of passive devices, but rather use constant cross-sectional dimensions, with a shank width that is typically smaller than that of passive neural probes.

Recently, a number of strategies have been proposed to exploit CMOS technology to construct the so called active dense tissue penetrating neural probes.

The NeuroSeeker probe (Raducanu et al., 2017) implements active electrodes with *in situ* circuits for signal amplification and supports simultaneous recording of all 1356 electrodes by implementing a time division multiplexing strategy.

A similar strategy, based on an Active Pixel Sensor (APS) array with small front-end circuits located beneath each electrode (or electrode-pixel), was developed in the SiNAPS (Simultaneous Neural Recording Active Pixel Sensor) probe technology (Angotzi et al., 2019). The SiNAPS probes also integrate in-pixel low-pass filters, allowing to reduce noise components generated by time-division-multiplexing circuits that may fold into the electrode signals. SiNAPS probes with different layouts, number of shanks and electrodes, and different shank sizes have been fabricated (Ribeiro et al., 2022).

In contrast, the NeuroPixel probe (Jun et al., 2017) integrates an active switching strategy, which allows the selection of 384 of the 960 Titanium Nitride recording sites on a single 10 mm long, 70 μm by 20 μm cross section shank. Voltage signals from the selected recording sites are then filtered, amplified, multiplexed and digitalized on the base of the probe.

Sayed Herbawi et al. (2018) implemented an active CMOS probe with 1,600 recording sites and 32 analog output channels. In this case, an electronic depth control approach using a digital hierarchical addressing scheme was implemented to record from subsets of 32 out of the 1,600 electrodes. This strategy allows to perform recordings from multiple brain areas without the need for physically adjusting probe position and results in a pronounced reduction of the interconnection overhead.

Researchers at the University of Freiburg presented a technological solution for the incorporation of digitalization electronics within the shank (rather than on the base) of active CMOS probes. In this case, Analog-to-Digital Converter (ADC) circuits were directly integrated beneath each electrode site (De Dorigo et al., 2018; Wendler et al., 2023). Moving the whole signal conditioning and acquisition circuits in the probe shank allows to significantly reduce the dimensions of the base and the total number of required interconnection wires (minimum of 7). This has the advantage to simplify the realization of fully immersible free-floating sub-cortical probes, which are required for applications targeting deep brain regions. However, in these solutions, the integration of additional electronic circuits in the probe shank comes at the cost of a reduced spatial resolution and increased power density.

**Figure 8:**
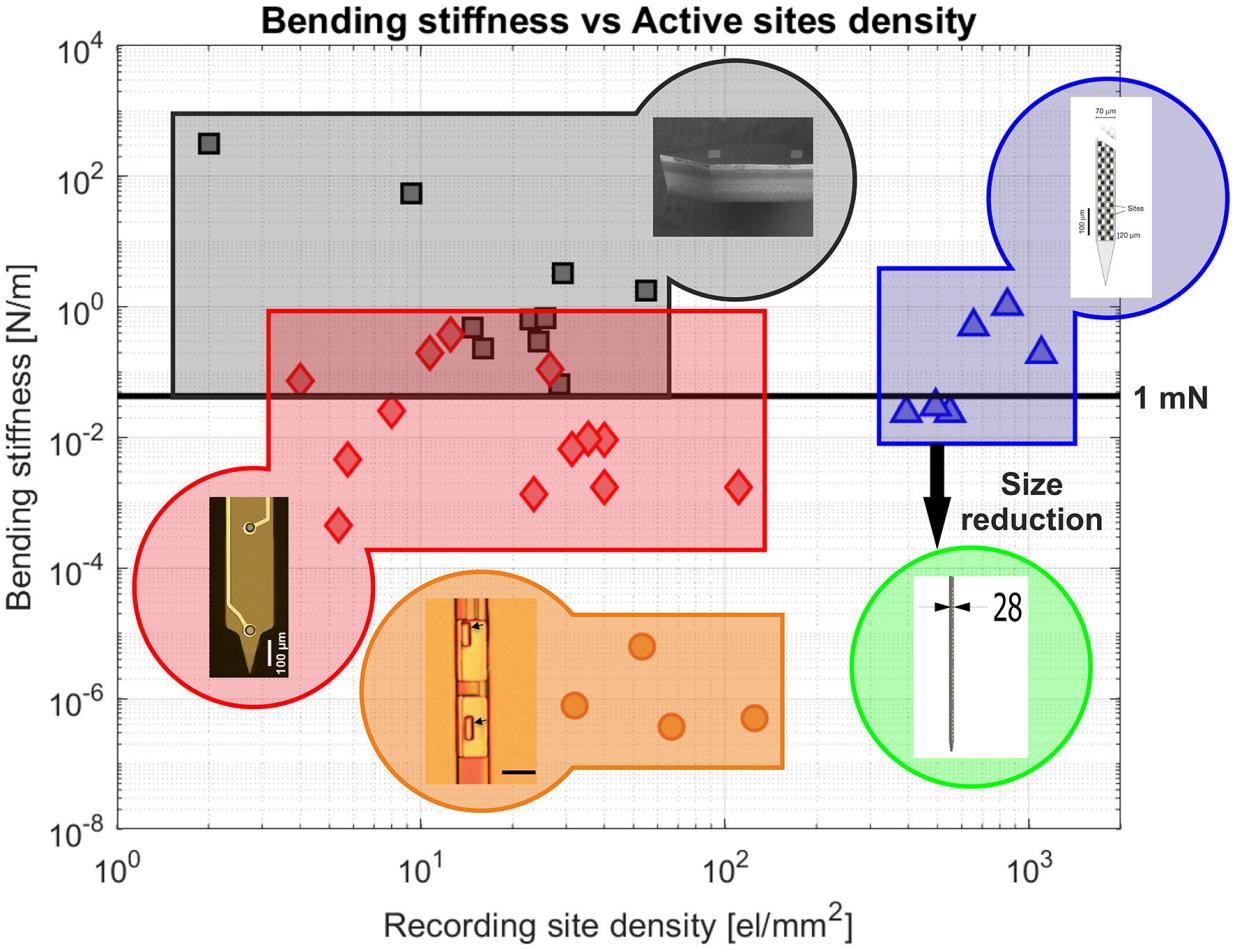
Summary plot of the relationship between the surface density of microelectrode sites and the bending stiffness for different types of neural probes. Black squares represent stiff passive silicon probes (representative image adapted from Fekete, 2015), red diamonds represent passive flexible probes (image adapted from Srikantharajah et al., 2021), orange circles represent ultra-flexible passive probes (image adapted from Luan et al., 2017) and blue triangles represent active CMOS probes (image adapted from Jun et al., 2017). The green area illustrates the proposed targets for the next-generation of intracortical neural probes for BCIs. The targeted device shall combine an high density and large number of microelectrode sites with low bending stiffness properties. The horizontal line represents the bending stiffness values that allow probes to withstand a penetration force of 1 mN without buckling. A threshold of 1 mN was chosen as a conservative force value allowing autonomous brain penetration. Polymeric probes display a significantly lower bending stiffness compared to silicon probes, but generally require an external aid to penetrate brain tissue. CMOS probes allow reaching a much higher electrode density than passive probes. The rectangle in yellow highlights ultra-flexible probes approaching a flexibility similar to that of brain tissue. Both axes are in a logarithmic scale. Sources: Drake et al. (1988), Rousche et al. (2001), Vetter et al. (2004), Herwik et al. (2009), Wester et al. (2009), Royer et al. (2010), Seidl et al. (2010), Winslow et al. (2010), Wu et al. (2011, 2015), Altuna et al. (2013), Xiang et al. (2014), Lopez et al. (2016), Raducanu et al. (2016), Jun et al. (2017), Luan et al. (2017), Angotzi et al. (2019), Chung et al. (2019), Musk (2019), Zatonyi et al. (2019), Scholten et al. (2020), Wang et al. (2020), Pimenta et al. (2021), Srikantharajah et al. (2021), and Cointe et al. (2022).

So far, the edge provided by CMOS technology has mainly been exploited to increase the density of electrodes on relatively large probes. However, CMOS technology also introduces the possibility of scaling down the size of intracortical neural probes, while maintaining a high surface density of recording sites, as illustrated in Figure 8. This approach is expected to become particularly beneficial when approaching cellular and sub-cellular cross-sectional dimensions. The CMOS technology nodes currently used for the fabrication of active neural probes (e.g., 180 nm node for SiNAPS and 130 nm node for NeuroPixel) already allow to reach cellular-like cross-sectional dimensions (below 30 μ), while the use of higher resolution nodes could open up the possibility of scaling down probes to sub-cellular dimensions. Moreover, despite the relatively low bending stiffness that these small silicon based devices would attain, they may be able to penetrate brain tissue autonomously, as insertion force has been shown to scale down with cross-sectional dimension (Obaid et al., 2020b).

Despite their advantages, CMOS-based neural probes also present challenges. The use of standard processes, as CMOS technologies, places strict restrictions on probe design and materials. Due to these restrictions, the CMOS wafer requires post-processing in order to convert the probe/tissue interface into biocompatible and brain resilient materials. The CMOS wafer also requires structuring in order to obtain the final probe shape, commonly by using dry-etching processes. Because CMOS electronics are embedded in the wafer, there are limitations on the minimum thickness of the probe shank, e.g., in the case of 0.18 μm technology around 10–11 μm (Tseng, 2022). Although neural probes are typically thicker, this technological limitation currently prevents the development of ultra-flexible monolithic CMOS probes.

Another challenging aspect which needs to be carefully considered during the design and testing of active CMOS probes is the power consumption and consequent tissue heating that may be generated. Indeed it is generally agreed that the temperature increase generated by invasive medical devices implanted in the brain should not exceed 1 K above brain temperature (Kim et al., 2007). Although this consideration also applies to passive neural probes, it is particularly relevant for active devices, because of the electronic circuits implemented in close contact with brain tissue and for which effective dissipation of heat toward the outside is more challenging. Raducanu et al. (2017) performed a FEM simulation of the heating induced by the NeuroSeeker probe and estimated a power dissipation limit of 4.5 mW for the implanted shank and 45 mW for the base in order to remain below the 1 K temperature increase threshold. Lopez et al. (2017) carried out a similar investigation for the NeuroPixel probe and came up with more stringent power consumption constraints of 1.8 mW for the shank and 20 mW for the base. Both studies highlight a stricter constraint on the power consumption of the probe shank compared to the base, potentially due to its high aspect ratio and due to it being in direct contact with brain tissue. This observation is particularly relevant for the design of active CMOS probes aiming to implement the whole signal processing chain in the shank of the probe (De Dorigo et al., 2018; Wendler et al., 2023), where it is most critical to assess and constrain power consumption to avoid excessive heating.

## 5. Discussion and perspective of prototype probe for chronic large-scale high resolution BCIs

Based on the literature discussed above, a few key aspects emerge which have the potential to guide advances in chronically implantable neural probes, in order to direct the chronic interaction between neural probes and brain tissue toward a low FBR. Based on the aspects emerging from this review, we propose a prototype device to achieve a long-lasting, high resolution and high channel count brain interfacing that could be used for next-generation BCIs.

Reducing the size of the neural probe shank can produce a more favorable integration of the device within the brain tissue. This stems from different mechanisms, particularly when approaching cross-sectional dimensions close to the size of cells. Size reduction is therefore a key strategy for achieving optimal probe-tissue integration. However, reducing the size of passive probes strongly limits the number of electrodes for each shank, and thus the number of simultaneously interfaced neurons as required for next generation BCIs.

Using passive probes, this can be overcome by implanting multiple probes, but at the cost of a larger number of tissue damaged sites, a strategy that, for instance, Neuralink has adopted (Musk, 2019).

A potential alternative solution is the use of active CMOS probes to achieve a high electrode density and an elevated channel count on each shank of the neural probes, as well as a low surface area. This strategy, however, imposes a constraint on the choice of the substrate material for the fabrication of the probe, limiting it to silicon. Silicon is a stiff material (170 GPa), but previous studies reviewed here have shown that the reduction in size is associated with a significant reduction in the overall device bending stiffness, which appears to be a more significant predictor of tissue response compared to substrate mechanical properties alone.

Furthermore, the packaging of the device is another key aspect driving mechanical interactions between the device and brain tissue. Devices which are rigidly tethered to the skull are subjected to a larger relative micromotion with brain tissue, resulting in stronger inflammation and FBR. To avoid this, it is important that the device is either tethered with a flexible interconnecting cable or, better yet, that it embeds wireless circuits for data communication and powering, although additional tissue heating generated through this approach should be carefully estimated and assessed to evaluate safety (Moon et al., 2021). The feasibility of interfacing active silicon probes with flexible polyimide interconnects to mechanically decouple stiff probes from the skull was proved by Barz et al. (2020). A different approach for achieving flexible interconnection of active CMOS probes with external electronics was presented by De Dorigo et al. (2018) and Wendler et al. (2023), who produced active probes with ADC circuits directly integrated in the probe shank rather than in the base. This strategy allows to reduce the required number of interconnection wires down to 7 and to shrink base dimensions, allowing to achieve free-floating probes with a high potential in applications involving subcortical deep brain regions.

Wireless circuits for data communication adapted to high channel count probes have been also reported (Crepaldi et al., 2018), and strategies for the wireless powering of neuro-devices that can be implanted and left floating inside the brain, such as the Microbead (Khalifa et al., 2019) or microsystems developed for organoid experimental models (Angotzi et al., 2022) are potential candidates to improve chronic reliability of electrical recordings and are currently under development, e.g., in the Crossbrain EIC project (CrossBrain, 2023).

Finally, it is also important to consider the tuning of the surface physicochemical properties of the device to further improve the long-term performance of chronic implants. A potential approach consists of coating the surface of the probe with highly hydrophilic polymers (Lu et al., 2009; Kozai et al., 2012b; Rao et al., 2012; Gutowski et al., 2015) or with zwitterionic molecules (Golabchi et al., 2019; Zou et al., 2021) to avoid the non-specific adsorption of protein on the surface of the probe, which is a driver of microglia/macrophages recruitment and activation of FBR processes. This strategy can also be combined with bioactive functionalizations to improve neuronal survival and discourage glial encapsulation.

In order to evaluate the effectiveness of different design strategies aiming to improve the chronic electrical interfacing performances of neural probes, it is of utmost importance to also establish standard testing protocols that closely mimic real-world device operating conditions. A wide-spread adoption of such testing methods, implementing clear and predefined objective metrics, would greatly improve the efficiency with which technological innovation can be translated into pre-clinical and clinical practice.

**Figure 9:**
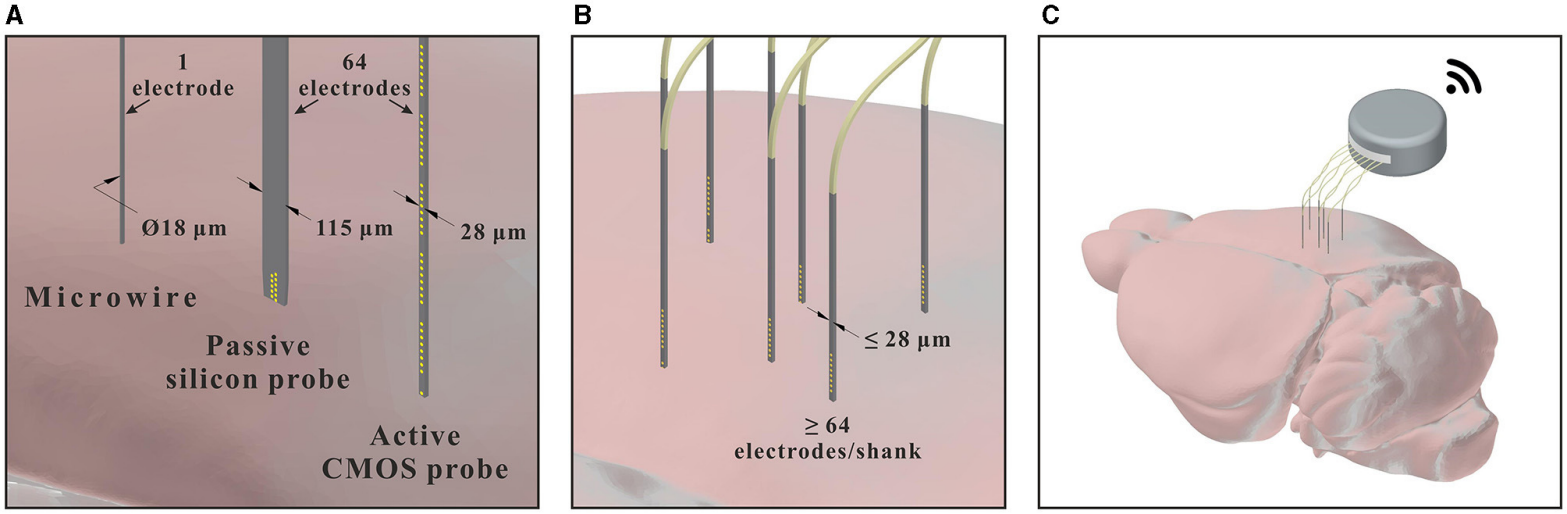
Proposed prototype for high quality chronic electrophysiological recordings. **(A)** Comparison of the size and electrode density for different neural probes. Microwires (left) can be fabricated with very small footprints but only have an individual electrode at their tip (18 μm diameter was selected based on Paradromics technology presented in Sahasrabuddhe et al., 2021). Passive silicon probes (middle) allow to place multiple electrodes on the surface of the shank but electrode density is limited by the requirement to individually route electrode sites to external electronics (A1x64-Poly2-6mm-23s-160 passive probe from NeuroNexus). Finally, active CMOS probes (right) allow to simultaneously scale dimensions down to microwire-like sizes while keeping a high channel count. The dimensions and electrode count in this example were inspired from the ChroMOS probes (Ribeiro et al., 2022). Despite the difference in their cross-sectional dimensions, both the passive and active silicon probes illustrated in **(A)** have 64 electrodes. **(B)** Image showing multiple high density (range of 1,000 electrodes/mm^2^) active devices implanted in a mouse brain [zoom-in from image **(C)**] and **(C)** Scheme of the proposed prototype device comprising data processing/transmission modules embedded in an implantable metal case and active CMOS probes with small cross-sectional size (i.e., below 30 μm, in the range of cellular dimensions) connected to external electronics via flexible leads.

The ideal neural recording device proposed here for long lasting, high channel count BCI applications is a high-density active CMOS probe with cross-sectional dimensions in the range of neuronal sizes, mechanically decoupled from the skull and equipped with a surface coating devised to reduce immune reaction. A packaging that allows simultaneous use of multiple such devices to target different brain regions and to further increase the channel count may also be necessary (as shown in Figures 9A–C). However, we anticipate that the number of total probes that need to be implanted to achieve high performing BCIs will most likely be much lower than what it is currently needed with passive devices.

In addition to the proposed design specifications for an ideal BCI candidate, the surgical implantation procedure should also be carefully tailored to minimize acute cortical and neurovascular damage.

In this respect, the measurement of insertion forces during device implantation may constitute an effective metric to assess the impact of both probe design (size and tip shape) and implantation protocol (e.g., insertion speed) on acute tissue damage. Closed loop implantation systems exploiting such a metric could increase safety and minimize the invasiveness of the implant. In fact, the insertion force could be used as a control signal to guide the insertion procedure and minimize implantation damage.

## Author contributions

AP: Conceptualization, Visualization, Writing — original draft, Writing — review & editing. GA: Writing — review & editing, Supervision. LB: Conceptualization, Funding acquisition, Supervision, Visualization, Writing — review & editing. JR: Conceptualization, Supervision, Writing — review & editing, Funding acquisition, Visualization.
